# Functional characterization of Fur in iron metabolism, oxidative stress resistance and virulence of *Riemerella anatipestifer*

**DOI:** 10.1186/s13567-021-00919-9

**Published:** 2021-03-19

**Authors:** Mi Huang, Mafeng Liu, Jiajun Liu, Dekang Zhu, Qianying Tang, Renyong Jia, Shun Chen, Xinxin Zhao, Qiao Yang, Ying Wu, Shaqiu Zhang, Juan Huang, Xumin Ou, Sai Mao, Qun Gao, Di Sun, Mingshu Wang, Anchun Cheng

**Affiliations:** 1 Institute of Preventive Veterinary Medicine, College of Veterinary Medicine of Sichuan Agricultural University, Chengdu, 611130 Sichuan China; 2 Research Centre of Avian Disease, College of Veterinary Medicine of Sichuan Agricultural University, Chengdu, 611130 Sichuan China; 3 Key Laboratory of Animal Disease and Human Health of Sichuan Province, Chengdu, 611130 Sichuan China

**Keywords:** Fur, Iron metabolism, Oxidative stress, Virulence, *Riemerella anatipestifer*

## Abstract

Iron is essential for most bacteria to survive, but excessive iron leads to damage by the Fenton reaction. Therefore, the concentration of intracellular free iron must be strictly controlled in bacteria. *Riemerella anatipestifer* (*R. anatipestifer*), a Gram-negative bacterium, encodes the iron uptake system. However, the iron homeostasis mechanism remains largely unknown. In this study, it was shown that compared with the wild type *R. anatipestifer* CH-1, *R. anatipestifer* CH-1Δ*fur* was more sensitive to streptonigrin, and this effect was alleviated when the bacteria were cultured in iron-depleted medium, suggesting that the *fur* mutant led to excess iron accumulation inside cells. Similarly, compared with *R. anatipestifer* CH-1*∆recA*, *R. anatipestifer* CH-1*∆recA*Δ*fur* was more sensitive to H_2_O_2_-induced oxidative stress when the bacteria were grown in iron-rich medium rather than iron-depleted medium. Accordingly, it was shown that *R. anatipestifer* CH-1*∆recA*Δ*fur* produced more intracellular ROS than *R. anatipestifer* CH-1*∆recA* in iron-rich medium. Electrophoretic mobility shift assays showed that *R. anatipestifer* CH-1 Fur suppressed the transcription of putative iron uptake genes through binding to their promoter regions. Finally, it was shown that compared with the wild type, *R. anatipestifer* CH-1Δ*fur* was significantly attenuated in ducklings and that the colonization ability of *R. anatipestifer* CH-1Δ*fur* in various tissues or organs was decreased. All these results suggested that Fur is important for iron homeostasis in *R. anatipestifer* and its pathogenic mechanism.

**Supplementary Information:**

The online version contains supplementary material available at 10.1186/s13567-021-00919-9.

## Introduction

Iron is an essential element for most organisms. In bacteria, iron is involved in several key metabolic processes, including respiration, tricarboxylic acid (TCA) cycling, oxygen transport, oxidative stress resistance, and DNA synthesis [1–4]. To survive, bacteria have evolved various systems to obtain iron from environment and/or host sources. However, excessive free iron in bacteria can produce toxic reactive oxygen species (ROS) and hydroxyl radicals through the Fenton reaction, which damage DNA, membranes and lipids [5, 6]. Therefore, bacteria developed scavenging systems (SOD isozymes, peroxidases, and catalases), and DNA repair systems (RecA, RecBCD, and RecF) to defend against oxidative stress [7–10]. RecA repairs oxidative DNA damage by combining with the RecF-like pathway or RecBCD pathway [8, 9]. In most Gram-negative bacteria, iron homeostasis is regulated by the ferric uptake regulator Fur [11–15]. When the concentration of intracellular free iron is high, the iron-associated Fur dimer binds to the promoter region of iron uptake genes to inhibit transcription, thus reducing iron intake. In contrast, when iron is deficient, iron dissociates from Fur, and Fur is released from the promoter region, leading to the increased transcription of iron uptake genes and thus increased iron intake [16].

*Riemerella anatipestifer* (*R. anatipestifer*), a Gram-negative bacterium belonging to the family *Flavobacteriaceae*, causes acute septicemia and infectious polyserositis in ducks, chickens, geese, and other avian species [17]. *R. anatipestifer* infection can give rise to high contagiousness and mortality in the duck industry [18], and at least 21 serotypes of *R. anatipestifer* without cross-protection have been identified [19, 20]. Besides, *R. anatipestifer* is resistant to multiple antibiotics [21–24], which is potentially related to its natural transformation ability [25]. Due to the presence of multiple serotypes and multidrug resistance, *R. anatipestifer* is hard to eradicate.

In previous studies, it has been shown that iron is essential for the survival of *R. anatipestifer* and that TonB plays an important role in iron and hemin uptake [26, 27]. Although genome analysis has shown that *R. anatipestifer* encodes putative iron uptake genes, the functions of most of them remain unclear [28]. It was shown that the putative iron-related TonB-dependent receptors B739_1208 and B739_1343 were important for pathogenesis, although they were not regulated by iron [29, 30]. Recently, it was found that some genes were significantly up-regulated under iron-limited conditions [31]. Although it has been shown that Fur of *R. anatipestifer* YM has a role in gene regulation, the role of Fur in iron homeostasis, oxidative stress resistance, and pathogenesis were not fully understood [32]. In this study, the role of *R. anatipestifer* CH-1 Fur in maintaining iron homeostasis, oxidative stress resistance, and pathogenesis were investigated.

## Materials and methods

### Bacterial strains, plasmids, and primers

The strains and plasmids used in this study are listed in Additional file 1. The primers used in this study are listed in Additional file 2.

### Growth conditions

*R. anatipestifer* strains were grown routinely on LB agar supplemented with 5% sheep blood or in GCB liquid medium [25] at 37 °C with shaking. Iron-rich and iron-limited conditions were achieved by GCB medium and GCB medium supplemented with different concentrations of ethylenediamine-di-o-hydroxyphenylacetic acid (EDDHA) (Alfa chemistry, ACM1170021), respectively.

### Construction of the *R. anatipestifer* CH-1Δ*recA*, *R. anatipestifer* CH-1Δ*recA*Δ*fur* and *R. anatipestifer* CH-1Δ*recA*Δ*fur* pLMF03::*fur* complementation strains

Deletion of the genes was performed according to the natural transformation-based knockout method described in a previous study [25]. Briefly, the up- and downstream sequence of *R. anatipestifer* CH-1 *recA* were amplified by PCR using the primers listed in Additional file 2. The sequence containing the Cmp cassette was amplified from the *R. anatipestifer* CH-2 strain using primers CmpP1 and CmpP2 (Additional file 2). The PCR fragments (upstream, Cmp cassette, and downstream) were ligated using the overlap PCR method. The fused PCR fragments were purified and incubated with *R. anatipestifer* CH-1 for 1 h at 37 °C. Then, samples of the mixture were spread onto plates supplemented with chloramphenicol and incubated overnight at 37 °C. The correct clone was identified as described in a previous study [33]. The mutant *R. anatipestifer* CH-1Δ*recA*Δ*fur* was constructed by the same method on the basis of *R. anatipestifer* CH-1 Δ*fur*, which was constructed in a previous study [33].

To construct the *R. anatipestifer* CH-1Δ*recA*Δ*fur*pLMF03::*fur* complementation strain, the plasmid pLMF03::*fur* was transformed into cells of the strain *Escherichia coli* S17-1, and the recombinant plasmid was introduced into the *R. anatipestifer* CH-1Δ*recA*Δ*fur* mutant strain via conjugation as described elsewhere [27]. The transconjugants were selected using blood agar plates supplemented with Cfx (1 μg/mL) and Kan (50 μg/mL) and identified by PCR amplification.

### Streptonigrin sensitivity assay

For indirect quantification of the intracellular iron level, we performed a streptonigrin sensitivity assay as described previously [34]. Briefly, *R. anatipestifer* CH-1pLMF03, *R. anatipestifer* CH-1Δ*fur*pLMF03 and *R. anatipestifer* CH-1Δ*fur*pLMF03::*fur* were grown to OD_600_ = 1.0 in GCB medium, GCB medium supplemented with 100 μM EDDHA, and GCB medium supplemented with 100 μM EDDHA and 200 μM Fe(NO_3_)_3_ at 37 °C in a shaking incubator. Cells were harvested by centrifugation at 6000 rpm for 10 min, and pellets were diluted with fresh PBS up to OD_600_ = 0.5 and aliquoted at 1 mL/tube. Streptonigrin (Sigma-Aldrich, St. Louis, USA) was diluted to 1 µg/mL with sterile PBS, 0 µL, 50 µL, and 80 µL was added to each tube of bacterial solution, the final concentration of streptonigrin was 0 ng/mL, 50 ng/mL and 80 ng/mL, respectively. Then the samples were incubated in the static incubator at 37 °C for 30 min. After incubation, the bacterial solution was diluted and spread onto GCB plates for counting (T0, T50, and T80). After a 1-day incubation at 37 °C, the grown colonies were counted. The survival rate was calculated as (T50/T0) × 100% and (T80/T0) × 100%, and the experiments were performed in triplicate.

### In vitro growth rate determination

The in vitro growth rates of the test strains were determined by measuring the OD_600_ with a spectrophotometer (Eppendorf Biophotometer, Germany). Briefly, *R. anatipestifer* CH-1pLMF03, *R. anatipestifer* CH-1Δ*fur*pLMF03 and *R. anatipestifer* CH-1Δ*fur*pLMF03::*fur* were cultured overnight and inoculated into 20 mL of GCB liquid medium at an OD_600_ of 0.05, and growth rates at 37 °C were determined by measuring the OD_600_ every 2 h for 12 h. In parallel, *R. anatipestifer* CH-1 and *R. anatipestifer* CH-1Δ*fur* were cultured overnight in iron-limited medium, then the overnight-cultured cells were subcultured into 20 mL of GCB or GCB supplemented with 50 μM EDDHA, 100 μM EDDHA or 200 μM EDDHA at an OD_600_ of 0.05, and growth rates were monitored by measuring OD_600_ as mentioned above. The data were analyzed using three independent experiments, with two replicate samples for each experiment.

### H_2_O_2_ sensitivity assay

A hydrogen peroxide (H_2_O_2_) challenge assay was performed as described in a previous study, with slight modification [35]. The sensitivity of the *fur* mutant to H_2_O_2_ was determined using a strain lacking *recA*, which is defective in DNA repair and thus more sensitive to H_2_O_2_ than the parent strain [36]. The strains *R. anatipestifer* CH-1Δ*recA* pLMF03, *R. anatipestifer* CH-1 Δ*recA*Δ*fur*pLMF03 and *R. anatipestifer* CH-1Δ*recA*Δ*fur*pLMF03::*fur* were grown in GCB liquid medium or GCB medium supplemented with EDDHA (25 μM or 50 μM) until the exponential phase (OD_600_ = 1.0–1.5). The cells were collected, washed and diluted in PBS to OD_600_ = 0.5, and aliquoted at 1 mL/tube. For the H_2_O_2_ challenge assay, each tube of the bacterial suspension was incubated with H_2_O_2_ (0, 5 or 10 mM) at 37 °C for 30 min. After exposure to H_2_O_2_, the bacteria were washed twice with PBS, and serial dilutions were spread onto GCB plates. After a 1-day incubation at 37 °C, the grown colonies were counted. The survival rate was calculated as described above, and the experiments were performed in triplicate.

### Fluorescence dye-based intracellular ROS detection

To detect intracellular ROS levels, the fluorescent reporter dye 5-(and-6)-chloromethyl-2′,7′-dichlorodihydrofluorescein diacetate, acetyl ester (CM-H_2_DCFDA, Life Technologies) was used. Briefly, the strains *R. anatipestifer* CH-1pLMF03, *R. anatipestifer* CH-1Δ*fur*pLMF03 and *R. anatipestifer* CH-1Δ*fur*pLMF03::*fur* were grown in GCB liquid medium or GCB supplemented with EDDHA (25 μM or 50 μM) until the exponential phase (OD_600_ = 1.0–1.5). Cells were collected and washed and diluted in PBS to OD_600_ = 0.5, and 1 mL samples were collected. Then, the samples were resuspended in 1 mL of PBS containing 10 μM CM-H_2_DCFDA. Samples were incubated in the dark for 30 min at room temperature. The cultures were precipitated by centrifugation; the supernatants were removed and then the cells were resuspended in 1 mL of PBS containing 5 mM H_2_O_2_ or 10 mM H_2_O_2_. After 30 min of treatment in the dark at 37 °C, the cell suspensions (200 μL) were transferred to a dark 96-well plate. Fluorescence signals were measured using a Varioskan Flash (Thermo Scientific) with excitation/emission wavelengths of 495/520 nm. Bacterial cells resuspended in sterile PBS were used as a negative control, 1 mL of PBS containing 10 μM CM-H_2_DCFDA and supplemented with 100 μM H_2_O_2_ was used as a positive control, and 1 mL of sterile PBS containing 10 μM CM-H_2_DCFDA was used as a black control. The incubation conditions were the same as those of the experimental groups, and the experiments were performed in triplicate.

### qRT-PCR

Real-time PCR was performed as described in a previous study [27]. Briefly, *R. anatipestifer* CH-1, *R. anatipestifer* CH-1Δ*fur* and *R. anatipestifer* CH-1Δ*fur*pLMF03::*fur* were grown in GCB or GCB supplemented with 100 μM EDDHA to exponential phase (OD_600_ = 1.0–1.5), and RNA was extracted by the RNeasy Minikit procedure (Qiagen). cDNA synthesis was performed with reverse transcriptase (HiScript Q RT SuperMix for qPCR gDNA wiper, R223-01, Vazyme, Nanjing, China). Real-time PCR was performed with SYBR Green master mix (Q111-03, Vazyme) using a CFX Connect real-time PCR detection system (Bio-Rad Laboratories, Hercules, CA, USA). Then, the transcription levels of TonB-dependent receptor genes *B739_0103* and *B739_0173* were detected in each sample using specific primers [33]. Relative fold changes were calculated as described previously with the threshold cycle (ΔΔCT) method, considering the efficiency of the PCR for each target [37]. Quantitative measurements were performed on biological samples in triplicate, and the results were normalized to findings with the *R. anatipestifer* housekeeping gene *recA* [27].

### Electrophoretic mobility shift assays (EMSAs)

DNA mobility shift assays were performed using the method described in a previous study, with minor modifications [38]. The promoter regions of the *B739_0173* and *B739_0173*-coding regions (204 bp and 214 bp, respectively) were amplified by PCR with the primers B739_0173 promoter P1/P2 and B739_0173 coding region P1/P2 (Additional file 2). Fifty to two hundred fifty nanograms of *B739_0173* promoter DNA or 250 ng of *B739_0173-*coding region DNA was mixed with 4 µg of Fur_6His_ protein in binding buffer (40 mM Tris–HCl, pH 8.0, 50 mM KCl, 2 mM DTT, 6% glycerol, 0.2 mM MnCl_2_ or 0.2 mM EDTA) in a 20 µL (final volume) mixture and incubated at 37 °C for 30 min. A 6% nondenaturing polyacrylamide gel in 0.5 × TBE running buffer was prerun for 30 min at 100 V and loaded with 20 µL of the binding reaction mixture. After being run for 2 h at 100 V, the gel was stained with Goldview and Coomassie Brilliant Blue.

### LD_50_ determination

The median lethal dose (LD_50_) was measured to evaluate virulence as previously described [30]. Briefly, *R. anatipestifer* CH-1, *R. anatipestifer* CH-1Δ*fur* and *R. anatipestifer* CH-1Δ*fur*pLMF03::*fur* were cultured in TSB medium at 37 °C with shaking until the exponential growth phase (OD_600_ = 1.0–1.5), and the bacteria were collected and washed and diluted in PBS. Each strain was prepared at the following doses: 5 × 10^10^ CFU/mL, 5 × 10^9^ CFU/mL, 5 × 10^8^ CFU/mL, and 5 × 10^7^ CFU/mL. Subsequently, the above doses of the bacteria were injected intramuscularly into the ducklings (10 ducklings/group), with each duckling receiving 0.2 mL. Once the ducklings exhibited signs of moribundity, they were euthanized via forced CO_2_ inhalation, and dead ducklings were subjected to *R. anatipestifer* identification by PCR and Gram staining. The mortality of the ducklings was recorded daily for 7 days post-challenge. The LD_50_ was calculated by using the Reed-Muench method [39].

### Colonization assays

To assess bacterial colonization ability in ducklings, 3-day-old ducklings were infected intramuscularly with *R. anatipestifer* CH-1pLMF03, *R. anatipestifer* CH-1Δ*fur*pLMF03 and *R. anatipestifer* CH-1Δ*fur*pLMF03::*fur* (10^9^ CFU/duckling). The initial bacterial number was estimated by OD_600_ and counted by spreading on blood plates. At 24 h and 48 h post-infection, six surviving ducklings in each test group were randomly selected and euthanized by forced CO_2_ inhalation. Liver, spleen, brain and blood from the heart were collected and weighed. The samples were homogenized in PBS (0.1 g of sample/0.9 mL of PBS) using a Nasco WHIRL–PAK (B01245WA, USA) as described previously [30]. The homogenized contents were serially diluted in PBS buffer and spread on blood agar plates supplemented with 50 μg/mL kanamycin to determine the bacterial CFU since *R. anatipestifer* is naturally resistant to kanamycin [26]. The plates were incubated at 37 °C overnight for counting and calculating the loads per gram of tissue [40].

### Duck serum bactericidal assay

Duck serum was obtained from the whole blood of 7-day-old ducklings via jugular vein bleeding. Blood samples were centrifuged twice (3500 rpm for 5 min) to obtain non-inactivated serum, and the serum was heat-inactivated at 55 °C for 1 h to obtain inactivated serum, which was stored at −20 °C before use. Bacterial survival in serum was determined as described in a previous study, with minor modifications [41]. Briefly, *R. anatipestifer* CH-1pLMF03, *R. anatipestifer* CH-1Δ*fur*pLMF03 and *R. anatipestifer* CH-1Δ*fur*pLMF03::*fur* were grown in GCB liquid medium to the exponential phase (OD_600_ = 1.0–1.5), the viable bacteria were washed twice with PBS, and the concentration of bacteria was adjusted to 10^9^ CFU/mL. Then, the mixture containing the cell suspension and 50% non-inactivated duck serum or 50% inactivated serum were incubated at 37 °C for 0.5 h and 1 h. The number of surviving bacteria was then determined by GCB plate counting. The survival rate was calculated as follows: the number of viable bacteria treated with non-inactivated duck serum or inactivated serum compared to the number of viable bacteria without treatment. The experiments were performed in triplicate.

### Statistical analysis

All experimental data are expressed as the mean ± 1 standard deviation (SD). Statistical analysis was performed using GraphPad Prism 7.00 (GraphPad Software, CA, USA) and SPSS Statistics 20 for Windows. The independent Student’s *t*-test was utilized to compare two groups, and one-way analysis of variance (ANOVA) or two-way ANOVA was used to compare multiple groups. *P* < 0.05 was considered significant.

## Results

### The *R. anatipestifer* CH-1 *fur* mutant was more sensitive to streptonigrin

**Figure 1:**
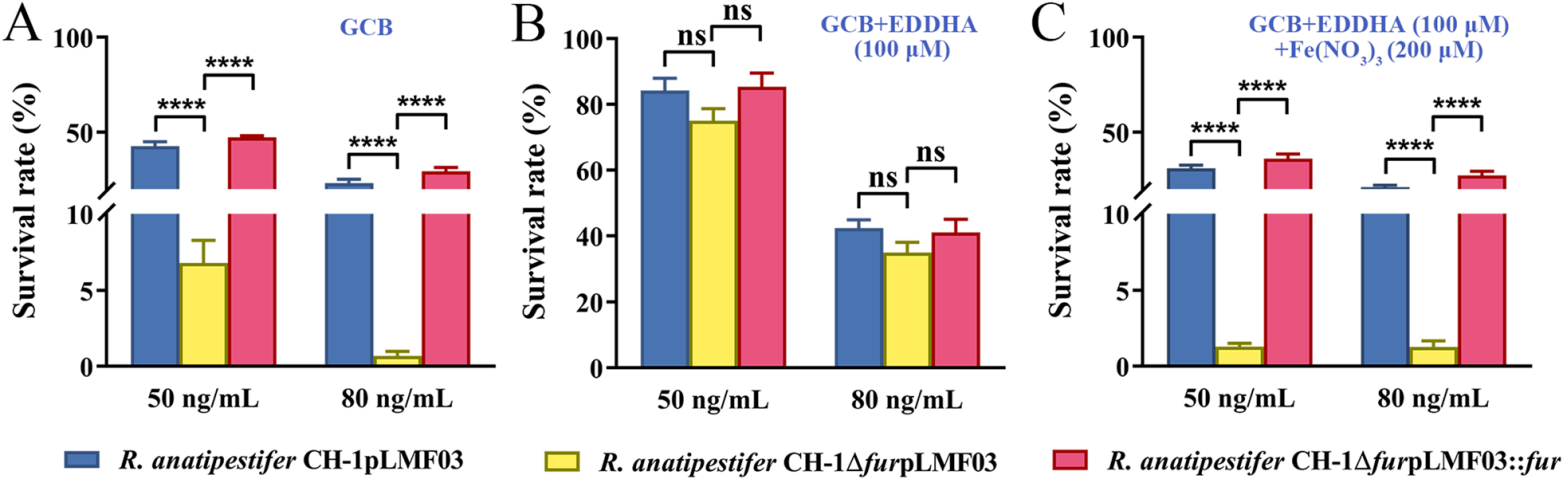
**The sensitivity of R. anatipestifer CH-1 and its fur mutant to streptonigrin.** The *R. anatipestifer* CH-1pLMF03, *R. anatipestifer* CH-1Δ*fur*pLMF03 and *R. anatipestifer* CH-1Δ*fur*pLMF03::*fur* strains were grown in GCB medium with no addition (**A**), with 100 μM EDDHA (**B**) or with 100 μM EDDHA and 200 μM Fe(NO_3_)_3_ (**C**) at 37 °C in a shaking incubator to the exponential phase (OD = 1.0–1.5). The bacteria were collected and suspended in PBS at 0.5 OD/mL, and were exposed to streptonigrin (50 ng/mL and 80 ng/mL) for 30 min. The survival rate of each culture was determined as described in the “Materials and methods”. The error bars represent the standard deviations of three independent experiments and three replicate samples for each experiment. Statistical significance was determined using two-way ANOVA (****P < 0.0001, ** *P* < 0.01, * *P* < 0.05).

To identify whether the *fur* mutation led to an increased intracellular free iron concentration in *R. anatipestifer* CH-1, we checked the sensitivity of the bacteria to streptonigrin since it is bactericidal in the presence of iron [34, 42]. Firstly, the bacteria were grown in iron rich medium, and the collected bacteria were used to measure the sensitivity to streptonigrin as described in “Materials and methods”. As shown in Figure 1A, the survival rate of *R. anatipestifer* CH-1Δ*fur*pLMF03 was approximately sevenfold lower than that of *R. anatipestifer* CH-1pLMF03 after treatment with 50 ng/mL streptonigrin. The survival rate of *R. anatipestifer* CH-1Δ*fur*pLMF03 was ~20-fold lower than that of *R. anatipestifer* CH-1pLMF03 when the concentration of streptonigrin was increased to 80 ng/mL. Moreover, the survival rate of the *fur* mutant strain was restored by the expression of Fur *in trans* (Figure 1A). To further verify that this effect was caused by iron, 100 μM the iron chelator EDDHA was added to the medium when the bacteria were cultured. Under this condition, the survival rates of all the strains were enhanced when treated with 50 ng/mL or 80 ng/mL streptonigrin. The survival rates of *R. anatipestifer* CH-1pLMF03, *R. anatipestifer* CH-1Δ*fur* pLMF03 and *R. anatipestifer* CH-1Δ*fur*pLMF03::*fur* were ~80%, ~75%, and ~80%, respectively, when treated with 50 ng/mL streptonigrin under iron-limited conditions (Figure 1B). In parallel, the survival rates of *R. anatipestifer* CH-1pLMF03, *R. anatipestifer* CH-1Δ*fur*pLMF03 and *R. anatipestifer* CH-1Δ*fur*pLMF03::*fur* were ~40%, ~35%, and ~40%, respectively, when treated with 80 ng/mL streptonigrin under iron-limited conditions (Figure 1B). Moreover, the sensitivity of all the strains to streptomycin was restored when iron(III) nitrate was added to the iron-limited medium (Figure 1C). These results suggest that Fur-deficient cells were strongly sensitive to streptonigrin, potentially due to excess iron inside the cells.

### The effect of the *fur* mutation on growth was not caused by excess iron in cells

**Figure 2:**
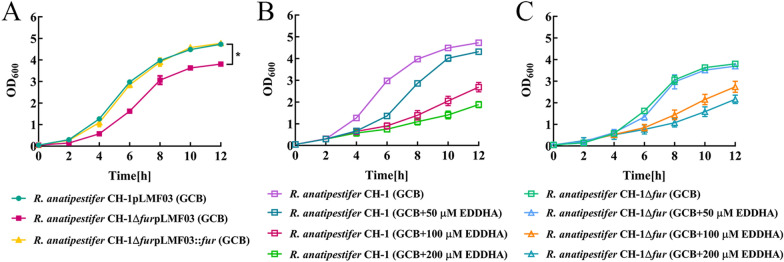
**The growth curves of R. anatipestifer CH-1 and its fur mutant.** **A** *R. anatipestifer* CH-1pLMF03, *R. anatipestifer* CH-1Δ*fur*pLMF03 and *R. anatipestifer* CH-1Δ*fur*pLMF03::*fur* cells were cultured overnight and then inoculated into 20 mL of GCB liquid medium at an OD_600_ of 0.05, the growth rates were determined at 37 °C with shaking cultivation (180 rpm) by measuring the OD_600_ every 2 h for 12 h. *R. anatipestifer* CH-1 (**B**) or *R. anatipestifer* CH-1Δ*fur* (**C**) was precultured in iron-limited medium overnight and then inoculated into 20 mL of GCB or GCB supplemented with 50 μM EDDHA, 100 μM EDDHA and 200 μM EDDHA at an OD_600_ of 0.05, and the growth rates were monitored as above. The error bars represent the standard deviations of three independent experiments and three replicate samples for each experiment. Statistical significance was determined using two-way ANOVA (****P < 0.0001, ** *P* < 0.01).

As Fur plays an important role in the global gene regulation, the deletion of *fur* may diminish bacterial growth, therefore, we first tested whether the absence of *fur* influences *R. anatipestifer* CH-1 growth. The results showed that the growth ability of the *fur* mutant was significantly decreased in GCB medium compared to that of the wild type (Figure 2A) and that it was restored by the expression of Fur in the mutant strain *in trans* (Figure 2A). To investigate whether the growth defects of Δ*fur* were caused by potential intracellular increased iron concentration, we measured the growth curve of *R. anatipestifer* CH-1 and *R. anatipestifer* CH-1Δ*fur* in GCB supplemented with the different concentration of iron chelator EDDHA. As shown in Figure 2B, the growth of *R. anatipestifer* CH-1 became slower when 50 μM, 100 μM or 200 μM was added to the medium. At the same time, the results showed that the addition of 50 μM, 100 μM or 200 μM EDDHA to the GCB medium did not benefit the growth of the *fur* mutant (Figure 2C). This result indicates that the effect of Fur mutation on growth is not caused by excess iron in cells.

### The *fur* mutant is more sensitive to H_2_O_2_-induced oxidative stress

**Figure 3:**
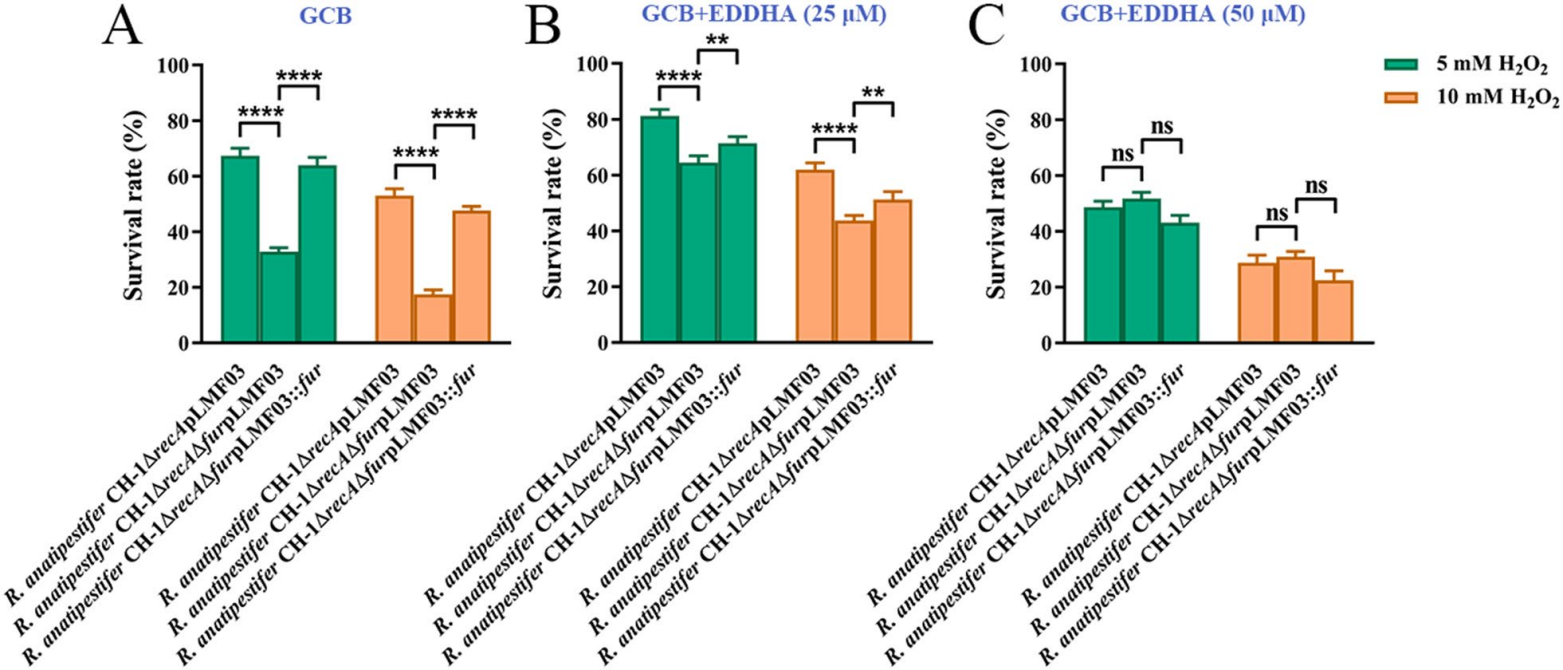
**The sensitivity of R. anatipestifer CH-1ΔrecA and its derived strains to H**_**2**_**O**_**2**_**-induced oxidative stress.** *R. anatipestifer* CH-1Δ*recA*pLMF03, *R. anatipestifer* CH-1Δ*recA*Δ*fur*pLMF03 and *R. anatipestifer* CH-1Δ*recA*Δ*fur*pLMF03::*fur* cells were cultured in GCB medium with no addition (**A**), with 25 μM EDDHA (**B**) or with 50 μM EDDHA (**C**) at 37 °C with shaking cultivation (180 rpm) to the exponential growth phase (OD_600_ = 1.0–1.5), and the cells were collected and diluted in PBS to 0.5 OD/mL. Then, the cell suspensions were incubated with 0 mM, 5 mM or 10 mM H_2_O_2_ at 37 °C for 30 min. The survival rate of each culture was determined as described in the “Materials and methods”. The error bars represent the standard deviations of three independent experiments and three replicate samples for each experiment. Statistical significance was determined using two-way ANOVA (****P < 0.0001, *** *P* < 0.001, ** *P* < 0.01).

Increased intracellular iron levels promote the decomposition of H_2_O_2_ and formation of hydroxyl radicals through the Fenton reaction, which damage cellular components [43]. The *fur* mutant was more sensitive to streptonigrin, which could be caused by increased intracellular iron concentrations. Thus, it was hypothesized that the *fur* mutant is more sensitive to H_2_O_2_. Compared with the wild type, the *fur* mutant did not display significantly increased sensitivity to H_2_O_2_ (data not shown). We then detected the sensitivity of the *fur* mutant strain to H_2_O_2_ in the *R. anatipestifer* CH-1 strain lacking *recA*, which is defective in DNA repair [36]. After exposure to 5 mM H_2_O_2_ and 10 mM H_2_O_2_, the survival rate of *R. anatipestifer* CH-1Δ*recA*Δ*fur* pLMF03 decreased significantly compared with that of *R. anatipestifer* CH-1Δ*recA*pLMF03 when cultured in GCB medium (Figure 3A). In parallel, we added different concentrations of EDDHA to GCB medium and then checked the sensitivity of these strains to H_2_O_2_. As Figure 3B shows, when the bacteria were cultured in GCB containing 25 μM EDDHA, the survival rate of *R. anatipestifer* CH-1Δ*recA*Δ*fur*pLMF03 was also decreased significantly compared with that of *R. anatipestifer* CH-1Δ*recA*pLMF03 when treated with 5 mM or 10 mM H_2_O_2_. However, when the bacteria were cultured in GCB containing 50 μM EDDHA, the survival rate of *R. anatipestifer* CH-1Δ*recA*Δ*fur*pLMF03 did not significantly decrease compared to that of *R. anatipestifer* CH-1Δ*recA*pLMF03 when treated with 5 mM and 10 mM H_2_O_2_ (Figure 3C). These results suggested that the deletion of *fur* in *R. anatipestifer* CH-1 significantly increased the sensitivity of this strain to H_2_O_2_ in iron-sufficient medium due to excess iron inside the cells.

### Deletion of *fur* causes increased intracellular ROS when treated with H_2_O_2_

**Figure 4:**
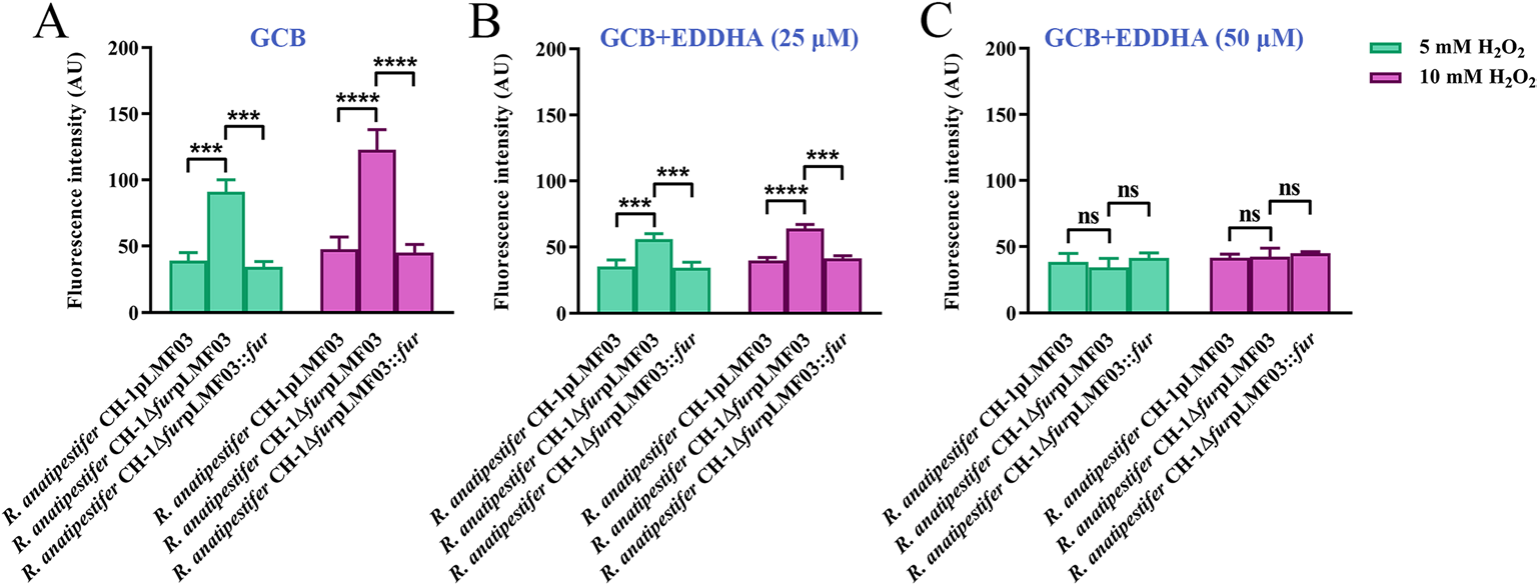
**Fluorescence-based ROS detection using the CM-H**_**2**_**DCFDA probe.** *R. anatipestifer* CH-1pLMF03, *R. anatipestifer* CH-1Δ*fur*pLMF03 and *R. anatipestifer* CH-1Δ*fur*pLMF03::*fur* cells were grown in GCB liquid medium with no addition (**A**), with 25 μM EDDHA (**B**) or with 50 μM EDDHA (**C**) at 37 °C in a shaking incubator until the exponential growth phase (OD_600_ = 1.0–1.5). Then, the cells were collected and diluted in PBS to 0.5 OD/mL, and the bacterial suspensions were treated with 10 μM CM-H_2_DCFDA for 30 min before treatment with 5 mM H_2_O_2_ or 10 mM H_2_O_2_ for 30 min in the dark. After exposure to H_2_O_2_, the cells from each culture were added to a dark 96-well plate, and the fluorescence signals were measured as described in the “Materials and methods”. The error bars represent the standard deviations of three independent experiments and three replicate samples for each experiment. Statistical significance was determined using two-way ANOVA (****P < 0.0001, *** *P* < 0.001).

Next, we determined if the absence of *fur* resulted in increased ROS in *R. anatipestifer* CH-1 in iron-rich medium after treatment with H_2_O_2_. The intracellular total ROS activity was measured by using CM-H_2_DCFDA, a permeability indicator of ROS [44]. Notably, after treatment with 5 mM H_2_O_2_, the fluorescence intensity of *R. anatipestifer* CH-1Δ*fur*pLMF03 was approximately 90 AU (absorbance unit), which was approximately twofold higher than that of *R. anatipestifer* CH-1pLMF03 in iron-rich medium, suggesting an increase in ROS in the *fur* deletion strain (Figure 4A). In parallel, we added different concentrations of EDDHA to GCB medium and checked the fluorescence intensity of these strains. When 25 µM EDDHA was added to the GCB medium, the fluorescence intensity of *R. anatipestifer* CH-1Δ*fur*pLMF03 was approximately 1.5-fold higher than that of *R. anatipestifer* CH-1pLMF03 (Figure 4B). When 50 µM EDDHA was added to the GCB medium, there was no difference in the fluorescence intensity between *R. anatipestifer* CH-1pLMF03 and *R. anatipestifer* CH-1Δ*fur*pLMF03 (Figure 4C). In addition, the fluorescence intensity of the *fur-*deficient strain could be restored by the expression of *fur in trans*. Overall, compared with that of the wild-type strain, the ROS content of the *fur* mutant strain increased in iron-sufficient medium when treated with H_2_O_2_.

### Fur binds to the promoters of putative iron uptake genes

**Figure 5:**
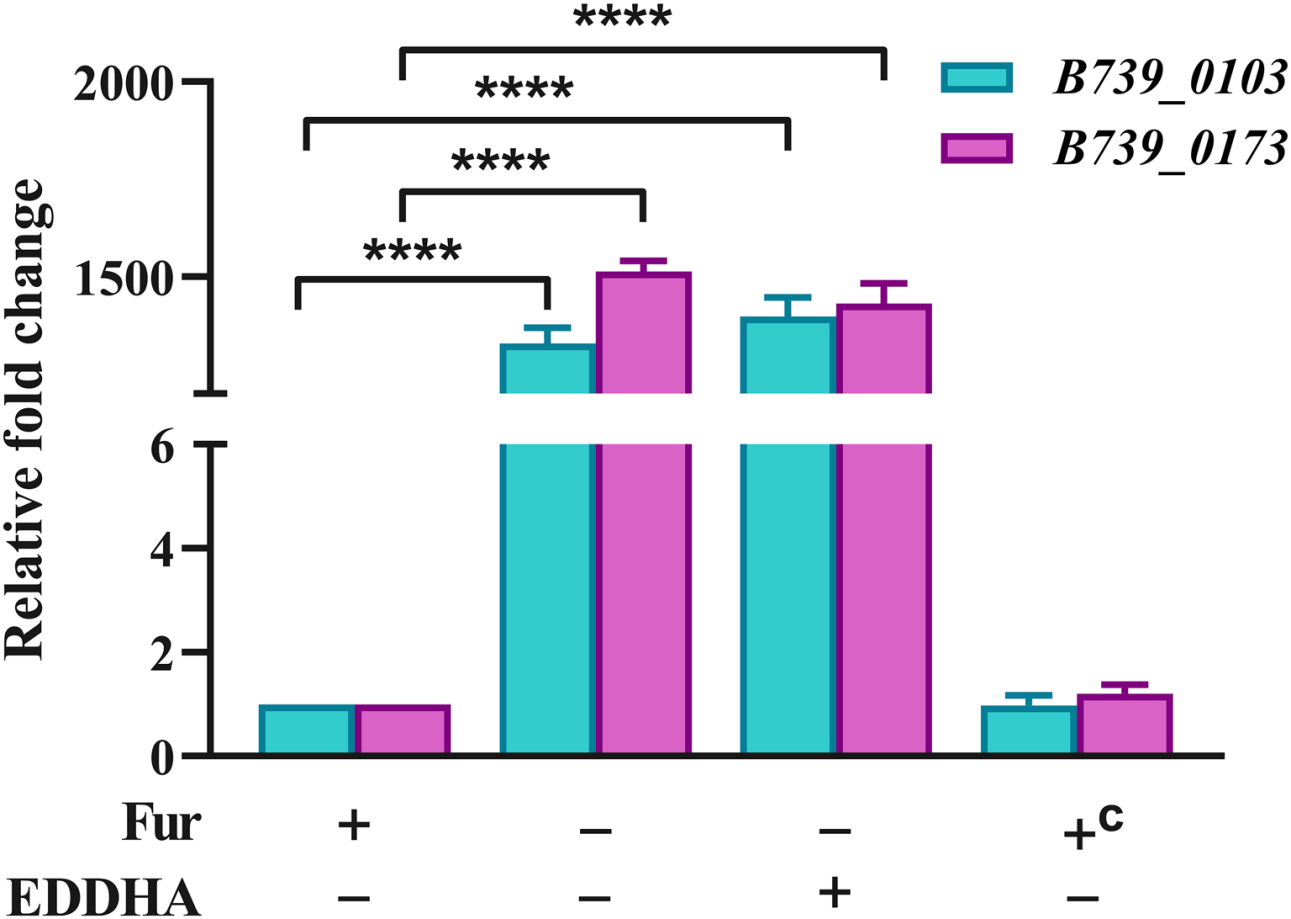
**Relative mRNA levels of B739_0103 and B739_0173 in R. anatipestifer CH-1 and its derived strains.** *R. anatipestifer* CH-1, *R. anatipestifer* CH-1Δ*fur* and *R. anatipestifer* CH-1Δ*fur*pLMF03::*fur* were grown in GCB or GCB supplemented with 100 μM EDDHA at 37 °C in a shaking incubator to the exponential growth phase (OD_600_ = 1.0–1.5). Total RNA and cDNA of these strains were prepared as described in the “Materials and methods”, and then the transcription of *B739_0103* and *B739_0173* was measured by qRT-PCR. Relative fold changes are reported in comparison with the parent strain. “c” means the complementary strain *R. anatipestifer* CH-1Δ*fur*pLMF03::*fur*. Statistical significance was determined using two-way ANOVA (****P < 0.0001).

The above results showed that the absence of *fur* led to a potentially increase in intracellular iron content, indicating that Fur is involved in the regulation of iron transport. Therefore, to verify whether *R. anatipestifer* CH-1 Fur can regulate the transcription of putative iron uptake-related genes, we detected the mRNA levels of TonB-dependent receptor genes *B739_0103* and *B739_0173*, which were up-regulated in an iron-limited environment and considered iron uptake-related genes [31, 33]. The results showed that the transcription of *B739_0103* and *B739_0173* was markedly increased in *R. anatipestifer* CH-1*∆fur* compared to that in the wild-type strain, and their transcript levels were not affected when 100 µM EDDHA was added to the GCB medium (Figure 5). Moreover, the increased transcription was fully restored to the wild-type level by the complementation of *fur* (Figure 5). These results indicated that Fur inhibits the transcription of the iron uptake genes *B739_0103* and *B739_0173* in *R. anatipestifer* CH-1.

**Figure 6:**
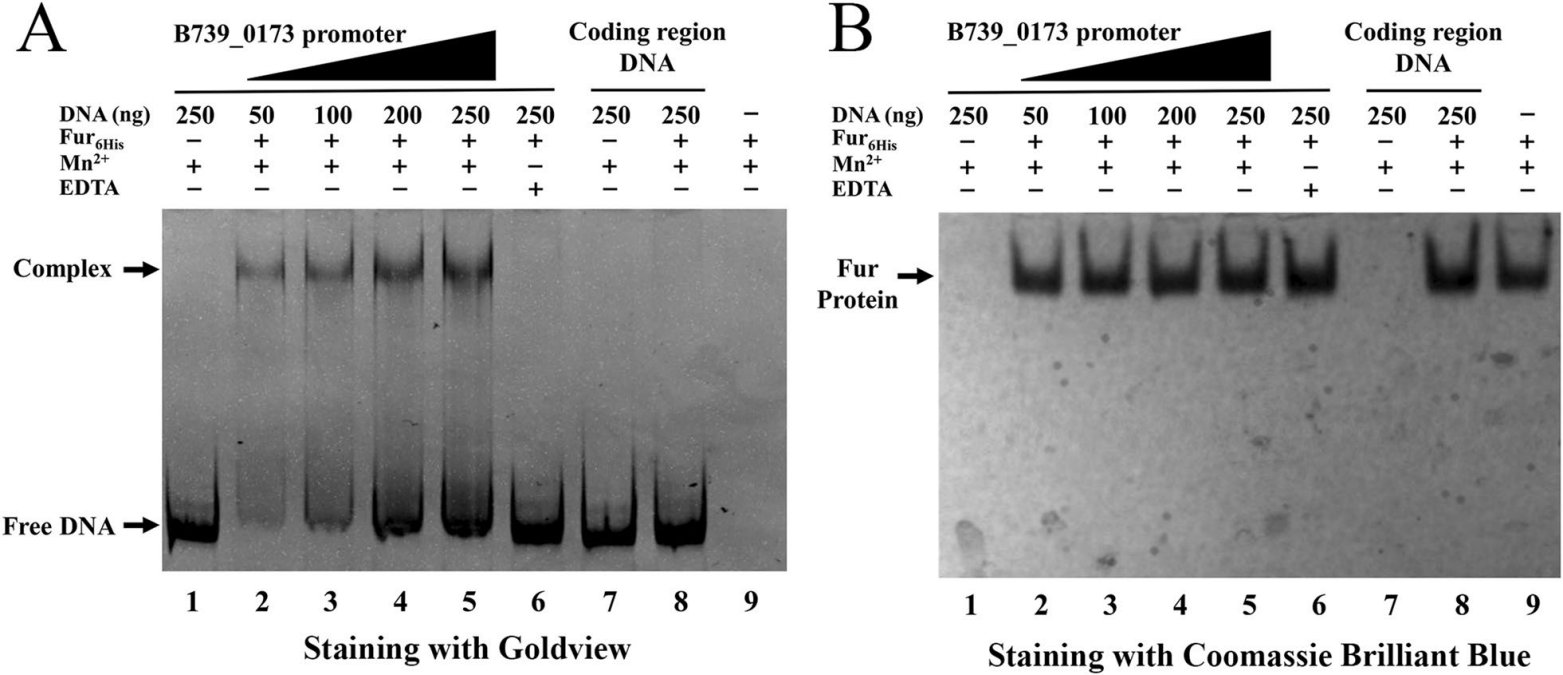
**Electrophoretic mobility shift assay (EMSA) for recombinant Fur**_**6His**_** binding with the promoter of B739_0173.** The DNA of the *B739_0173* promoter region and *B739_0173*-coding region were amplified by PCR, and 50–250 ng of *B739_0173* promoter DNA or 250 ng of *B739_0173-*coding region DNA was mixed with 4 µg of Fur_6His_ protein in binding buffer at 37 °C for 30 min. The samples were electrophoresed on a gel as described in the “Materials and methods”. (**A**) The gel was stained with Goldview. (**B**) The gel was stained with Coomassie Brilliant Blue. The experiment was repeated three times, the *B739_0103* promoter region with Fur_6His_ showed similar results, and a representative image is shown.

To explore how Fur regulates iron uptake genes, EMSAs were performed as described in the “Materials and methods”. Since Mn^2+^ has more stable chemical properties than Fe^2+^, it is a typical surrogate for iron to maintain the regulatory activity of Fur [45]. As shown in Figure 6, only in the reaction buffer containing 200 µM MnCl_2_, the incubation of the promoter region of the putative iron uptake gene *B739_0173* with purified Fur_6His_ led to the formation of DNA–protein complexes, which showed clearly retarded migration in the gels, and the complex formation was increased with higher DNA concentrations. The results suggest that Fur binds to the promoter region of target genes and that binding occurs only in the presence of Mn^2+^. As a negative control, the DNA fragment of the coding region did not form a complex with Fur_6His_ (Figure 6). Taken together, these results provide evidence that Fur inhibits the transcription of iron uptake-related genes by binding to the promoter region of these genes in *R. anatipestifer* CH-1.

### Fur contributes to the virulence and colonization ability of *R. anatipestifer* CH-1

In our previous study, we demonstrated that a *fur* deletion strain of *R. anatipestifer* showed reduced virulence in the *Galleria mellonella* model [33]. To investigate if Fur plays a role in the pathogenesis of *R. anatipestifer* CH-1 in poultry, we used a duckling model [29, 30] to determine the LD_50_ of the *fur* mutant. The calculated LD_50_ value of *R. anatipestifer* CH-1Δ*fur* pLMF03 was greater than 10^12^ CFU, whereas the LD_50_ values of *R. anatipestifer* CH-1pLMF03 and the complementation strain were 10^8^ CFU and 10^9^ CFU, respectively. These results showed that the *fur* mutation led to reduced virulence of *R. anatipestifer* CH-1 in ducklings.

**Figure 7:**
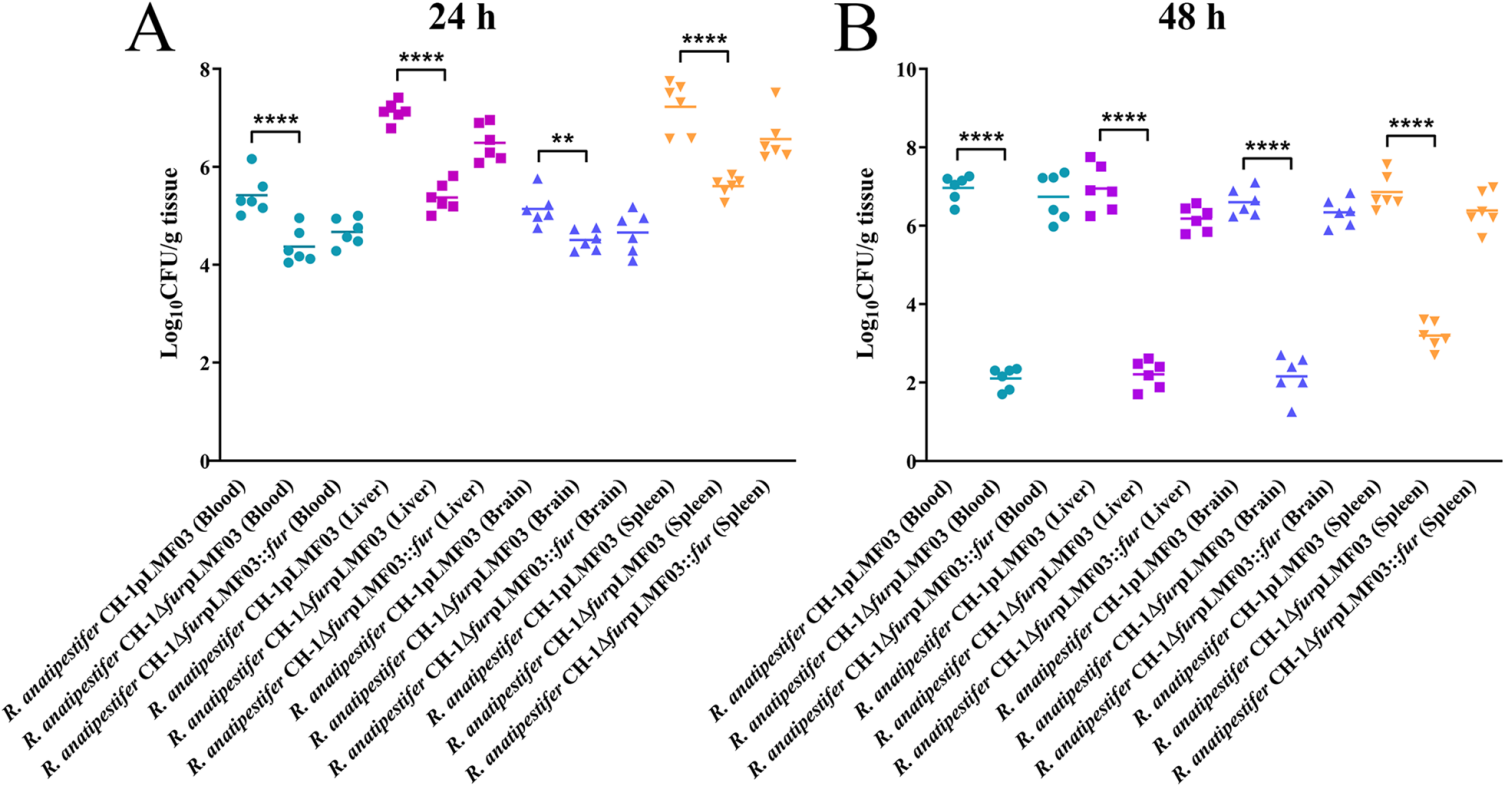
**Colonization of R. anatipestifer CH-1 and its fur mutant in ducklings at 24 h and 48 h post-infection.** Doses (200 μL) of 10^9^ CFU of *R. anatipestifer* CH-1pLMF03, *R. anatipestifer* CH-1Δ*fur*pLMF03 and *R. anatipestifer* CH-1 Δ*fur*pLMF03::*fur* were prepared and injected intramuscularly into 3-day-old ducklings (20 ducklings/group). At 24 h (**A**) and 48 h (**B**) post-infection, bacteria were isolated from the livers, spleens, brains, and the blood from the heart, as described in the “Materials and methods”. The data points represent the CFU/g values of the indicated organs in individual ducklings; the bars show the mean values (*n* = 6). Statistical significance was determined using two-way ANOVA (****P < 0.0001, ** *P* < 0.01).

To test whether the reduced virulence is due to a decrease in bacterial colonization ability, groups of ducklings were inoculated with 10^9^ CFU of *R. anatipestifer* CH-1pLMF03, *R. anatipestifer* CH-1Δ*fur*pLMF03 or *R. anatipestifer* CH-1Δ*fur*pLMF03::*fur* in the leg. Twenty-four hours and 48 h post-infection, the bacterial loads in the liver, spleen, brain, and the blood from the heart of the ducklings were determined. As shown in Figure 7A, at 24 h post-inoculation, the number of recovered colonies from various tissues and organs for the *R. anatipestifer* CH-1 *fur* mutant was significantly reduced compared to that of the parent strain (*P* < 0.0001) (Figure 7A). Similarly, at 48 h post-inoculation, the amount of colonized *R. anatipestifer* CH-1Δ*fur* in various tissues and organs was also significantly decreased compared to that of the parent strain (Figure 7B). Moreover, compared to 24 h post-infection, the gap between the *fur* mutant and the parent strain was increased (Figure 7B). In addition, the bacterial loads in each tissue of the complementation strain *R. anatipestifer* CH-1Δ*fur*pLMF03::*fur* at 24 h and 48 h were comparable to those of the wild-type strain (Figure 7). These results indicated that Fur not only contributes to the colonization of *R. anatipestifer* CH-1 in duckling tissues, such as the liver, spleen, brain, and the blood from the heart, but also protected *R. anatipestifer* CH-1 from host clearance.

### The *fur* mutant is susceptible to non-inactivated duck serum

**Figure 8:**
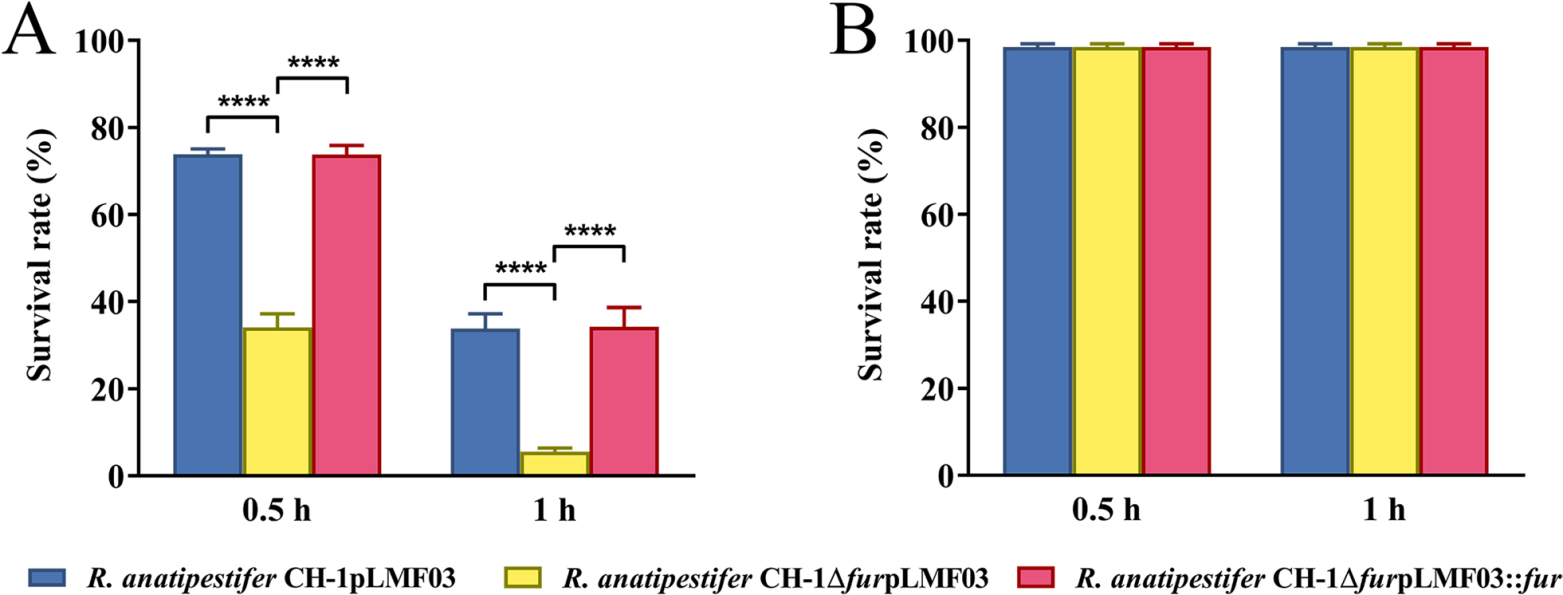
**The sensitivity of R. anatipestifer CH-1 and its derived strains to non-inactivated duck serum.** The non-inactivated duck serum and inactivated duck serum were obtained by centrifuging from the jugular vein-collected blood of 7-day-old ducklings as described in the “Materials and methods”. *R. anatipestifer* CH-1pLMF03, *R. anatipestifer* CH-1Δ*fur*pLMF03 and *R. anatipestifer* CH-1Δ*fur*pLMF03::*fur* were grown in GCB liquid medium at 37 °C in a shaking incubator to exponential phase (OD_600_ = 1.0–1.5). Cells were collected and diluted in PBS to 10^9^ CFU/mL, and the bacterial suspensions were incubated with 50% non-inactivated duck serum (**A**) or 50% inactivated duck serum (**B**) for 0.5 h or 1 h at 37 °C. Bacterial survival was enumerated by plating and counting colonies the following day as described in the “Materials and methods”. The error bars represent the standard deviations of three independent experiments and three replicate samples for each experiment. Statistical significance was determined using two-way ANOVA (****P < 0.0001).

Compared to that of wild type, *R. anatipestifer* CH-1Δ*fur* had a decreased colonization ability in blood. Therefore, it can be hypothesized that the *fur* mutant is susceptible to duck serum. To further investigate this hypothesis, the survival rates of *R. anatipestifer* CH-1pLMF03, *R. anatipestifer* CH-1Δ*fur*pLMF03 and *R. anatipestifer* CH-1Δ*fur*pLMF03::*fur* in 50% non-inactivated duck serum were measured. As shown in Figure 8A, after exposure to this serum for 0.5 h, the survival rate of *R. anatipestifer* CH-1 pLMF03 was ~70%, while the survival rate of *R. anatipestifer* CH-1Δ*fur* pLMF03 was significantly decreased compared to the parent strain. After incubation with 50% non-inactivated duck serum for 1 h, the survival rates of *R. anatipestifer* CH-1pLMF03, *R. anatipestifer* CH-1Δ*fur*pLMF03 and *R. anatipestifer* CH-1Δ*fur*pLMF03::*fur* were approximately 30%, 5% and 30%, respectively (Figure 8A). As a control, 50% inactivated duck serum had neither an effect on the survival of *R. anatipestifer* CH-1pLMF03 nor on the survival of *R. anatipestifer* CH-1Δ*fur*pLMF03, and there is no difference in survival rates among all strains when treated with 50% inactivated duck serum for 0.5 h or 1 h (Figure 8B). These results demonstrated that the lack of Fur has a detrimental effect on serum resistance, which may also lead to a decrease in the virulence of *R. anatipestifer* CH-1.

## Discussion

Iron is an essential element for the survival and growth of most bacteria; however, it can be toxic when present in excess [5, 6]. In some bacteria, iron inside bacterial cells is tightly regulated by the ferric uptake regulator Fur [46–48]. *R. anatipestifer*, an iron-dependent bacterium, has unclear mechanisms to regulate iron transport [32]. In the *R. anatipestifer* CH-1 genome, B739_0252 was annotated as a Fur family transcriptional regulator since it contains a Fur_like domain at amino acids 28–151. A protein BLAST analysis indicated that the Fur of *R. anatipestifer* CH-1 had low identity compared with well-characterized Fur proteins of other bacteria, such as *E. coli* (25% identity and 40% similarity), *Campylobacter jejuni* (25% identity and 39% similarity), and *Pseudomonas aeruginosa* (24% identity and 39% similarity). In this study, we determined the role of Fur in the physiology and virulence of *R. anatipestifer* CH-1.

Many studies have led to a classic model of Fur regulation in response to different iron conditions [46, 49, 50]. When the intracellular iron concentration is high, Fur-Fe^2+^ represses the expression of iron acquisition genes by binding upstream of these genes. When the intracellular iron concentration is low, Fur-Fe^2+^ dimers dissociate, which relieves the inhibition of iron acquisition genes, leading to an increased intracellular iron concentration. In this study, we found that the sensitivity of *R. anatipestifer* CH-1Δ*fur* to streptonigrin was significantly higher than that of *R. anatipestifer* CH-1, and the sensitivity was affected by the external iron concentrations. The antibiotic streptonigrin is bactericidal in the presence of iron, indicating that the lack of *fur* may cause an increase in free intracellular iron concentration in *R. anatipestifer* CH-1. Besides, in an iron-rich environment, the deletion of the *fur* gene could affect the growth of *R. anatipestifer* CH-1. Since the lack of Fur may increase the free intracellular iron concentration, it was hypothesized that the growth defect of the mutant strain is due to dysregulated iron acquisition. However, supplementation with different concentration of EDDHA in iron-rich medium did not improve the growth ability of the *fur* mutant, indicating that the growth defect of the *fur* mutant strain is not caused by an imbalance in iron uptake. This was not surprising, since in addition to iron metabolism regulation, Fur was also shown to be involved in other cellular processes as a global regulator [13, 48].

The inactivation of *fur* may lead to unrestrained iron uptake, thus leading to the accumulation of free iron in the cytoplasm when the bacteria are grown in iron-rich conditions. Finally, it will result in excessive iron-catalyzed production of ROS [51]. In this study, we also found that after *fur* deletion, the strain was more sensitive to H_2_O_2_ and increased levels of intracellular ROS could be detected. In summary, the higher susceptibility to streptonigrin and H_2_O_2_ and the accumulation of ROS in the Fur-deficient strain suggest that a key role of Fur in *R. anatipestifer* is to avoid iron intoxication and oxidative stress.

In our previous studies, it was shown that the putative TonB-dependent receptor genes *B739_0103* and *B739_0173* were up-regulated under iron-limited conditions [31], and this phenomenon prompted us to check whether this regulation relies on Fur in *R. anatipestifer* CH-1. Fur plays a role through binding to the promoter region of its target gene, and the putative Fur-box sequence (5′-GATAATGATAATCATTATC-3′) has been found in *R. anatipestifer* YM [1, 32, 52]. Sequence comparison showed that the sequence of the Fur box was also present in the promoter regions of *B739_0103* and *B739_0173*. As expected, it was shown that the transcription of *B739_0103* and *B739_0173* was significantly up-regulated in the *fur* mutant, suggesting that Fur may inhibit the transcription of iron uptake genes in *R. anatipestifer* CH-1. Moreover, it was shown that Fur was able to bind to the promoter region of *B739_0173* rather than the coding sequence in the presence of Mn^2+^. From these results, it can be concluded that *R. anatipestifer* CH-1 Fur is involved in regulating the transcription of iron uptake genes by binding to their promoters and that this process requires the participation of metal ions, which is different from the function of Fur in *Helicobacter pylori* and *C. jejuni*. In *H. pylori* and *C. jejuni*, Fur can form a dimer even without iron as a cofactor and directly bind to the promoter region of the target gene, which is called apo-Fur regulation [53–55].

The Fur protein contributes to virulence in animal models for numerous bacterial pathogens [32, 48, 56–60], but the precise mechanism of the attenuation of *fur* mutants is not completely clear. In *R. anatipestifer*, previous works identified that the absence of Fur could reduce virulence in ducklings and in *Galleria mellonella* larvae [32, 33]. In agreement with these studies, it was shown that the LD_50_ of the *fur*-deficient strain in ducklings was significantly higher (more than 10^4^ times) than that of the wild-type strain. The colonization ability of the *fur* mutant in ducklings was greatly diminished. Moreover, compared to the wild type, the *R. anatipestifer* CH-1*∆fur* mutant was more easily eliminated by the host.

As a mechanism of host defense against bacterial pathogen invasion, host innate immune cells, such as macrophages and neutrophils, produce superoxide radicals and hydrogen peroxide to kill invading bacteria [61]. Recent studies have shown that the host also uses iron or other metal toxicity at the site of infection to kill and control bacterial infection [62–64]. As antagonistic strategies, bacterial pathogens have evolved systems such as ROS detoxification, macromolecule damage repair, and metal efflux systems to survive in the host. Here, we can conclude that the decreased virulence of *R. anatipestifer* CH-1*∆fur* in ducks is partly due to its reduced resistance to oxidative stress. Moreover, we found that compared to the parent strain, the *fur* mutant was more easily killed by the non-inactivated duck serum*.* This supports the fact that *fur* deletion might lead to a decreased virulence of *R. anatipestifer* in ducks. It has been reported that a decrease in virulence of the *fur* mutant may be related to a reduction in the activity of enzymes required for protection against ROS, and changes in the expression of virulence factors in the *fur* mutant [12, 65]. Whether *R. anatipestifer* Fur regulates the expression of oxidative stress response enzymes and virulence genes needs to be investigated further. Regardless, the attenuated *R. anatipestifer* CH-1 *fur* mutant may provide the basis for future investigations of an attenuated vaccine. Overall, this study provides evidence of the essentiality of Fur in maintaining iron homeostasis, oxidative stress resistance and pathogenesis in *R. anatipestifer* CH-1.

## Supplementary Information

**Additional file 1. The bacterial strains and plasmids used in this study.**

**Additional file 2. The primers used in this study.**

## Data Availability

The nucleotide sequences of *R. anatipestifer* CH-1 were deposited in GenBank under accession number CP003787. The accession number of ferric uptake regulator Fur is following: Fur of *Riemerella anatipestifer* CH-1 (GenBank: AFR34859.1), Fur of *Escherichia coli* (GenBank: EFJ3478230.1), Fur of *Campylobacter jejuni* (GenBank: VTQ54023.1), Fur of *Pseudomonas aeruginosa* (GenBank: MXH36461.1). The datasets generated and/or analysed during the current study are available from the corresponding authors on reasonable request.

## Abbreviations

TCA: Tricarboxylic acid
ROS: Reactive oxygen species
GCB: Gonorrhoeae-culture broth
EDDHA: Ethylenediamine-N,N′-bis ((2-hydroxyphenyl) acetic acid)
H_2_O_2_: Hydrogen peroxide
OD_600_: Optical density at 600 nm
CM-H2DCFDA: 5-(And-6)-chloromethyl-2′,7′-dichlorodihydrofluorescein diacetate, acetyl ester
ΔΔCT: Threshold cycle
EMSA: Electrophoretic mobility shift assay
LD_50_: Median lethal dose
ANOVA: Analysis of variance
SD: Standard deviation
Amp: Ampicillin
Kan: Kanamycin
Cfx: Cefoxitin
