# Supplementary material for: Functional characterization of Fur in iron metabolism, oxidative stress resistance and virulence of Riemerella anatipestifer
Source: Vet Res. 2021 Mar 19;52:48. doi: 10.1186/s13567-021-00919-9 (PMC7976709; doi:10.1186/s13567-021-00919-9)
Supplement: Supplementary file 1 — Additional file 1. The bacterial strains and plasmids used in this study. [file 13567_2021_919_MOESM1_ESM.docx]

**Additional file 1** **The bacterial strains and plasmids used in this study.**

| *E. coli* **strains** | **Genotype or description** | **Source or reference** |
| --- | --- | --- |
| DH5α | F–Φ80lacZΔM15 Δ(lacZYA-argF) U169 recA1 endA1 hsdR17 (rK–, mK+) phoA supE44 λ– thi-1 gyrA96 relA1 | Laboratory collection |
| S17-1 | *hsdR17 recA1* RP4-2-tet::Mu-1kan::Tn7; Sm^R^ | Laboratory collection |
| ***Riemerella anatipestifer* strains** | **Genotype or description** | **Source or reference** |
| *R. anatipestifer* CH-1 | *R. anatipestifer* CH-1, Km^R^ | Laboratory collection |
| *R. anatipestifer* CH-2 | *R. anatipestifer* CH-2 | Laboratory collection |
| *R. anatipestifer* CH-1 pLMF03 | *R. anatipestifer* CH-1, pLMF03, Cfx^R^ | [30] |
| *R. anatipestifer* CH-1Δ*fur* | *R. anatipestifer* CH-1, *fur* mutant | [33] |
| *R. anatipestifer* CH-1Δ*fur* pLMF03 | *R. anatipestifer* CH-1, *fur* mutant, pLMF03, Cfx^R^ | This study |
| *R. anatipestifer* CH-1Δ*fur* pLMF03::*fur* | *R. anatipestifer* CH-1, *fur* mutant, pLMF03::*fur*, Cfx^R^ | This study |
| *R. anatipestifer* CH-1Δ*recA* | *R. anatipestifer* CH-1, *recA* mutant, Cmp^R^ | This study |
| *R. anatipestifer* CH-1Δ*recA*Δ*fur* | *R. anatipestifer* CH-1, *recA* mutant, *fur* mutant, Cmp^R^ | This study |
| *R. anatipestifer* CH-1Δ*recA*Δ*fur* pLMF03 | *R. anatipestifer* CH-1, *recA* mutant, *fur* mutant, pLMF03, Cmp^R^, Cfx^R^ | This study |
| *R. anatipestifer* CH-1Δ*recA*Δ*fur* pLMF03::*fur* | *R. anatipestifer* CH-1, *recA* mutant, *fur* mutant, pLMF03::*fur*, Cmp^R^, Cfx^R^ | This study |
| **Plasmids** | **Genotype or description** | **Source or reference** |
| pLMF03 | B739_0921 promoter, *ori*ColE1, *ori* pRA0726, Amp^R^, Cfx^R^ | [27] |
| pLMF03::*fur* | pLMF03 carrying fur from *R. anatipestifer* CH-1, Amp^R^, Cfx^R^ | This study |

Sm^R^, streptomycin resistance; Amp^R^, ampicillin resistance; Km^R^, kanamycin resistance; Cmp^R^, chloramphenicol resistance; Cfx^R^, cefoxitin resistance.
