# Supplementary material for: Functional characterization of Fur in iron metabolism, oxidative stress resistance and virulence of Riemerella anatipestifer
Source: Vet Res. 2021 Mar 19;52:48. doi: 10.1186/s13567-021-00919-9 (PMC7976709; doi:10.1186/s13567-021-00919-9)
Supplement: Supplementary file 2 — Additional file 2. The primers used in this study. [file 13567_2021_919_MOESM2_ESM.docx]

**Additional file 2** **The primers used in this study.**

| **Primer** | **Organism** | **Sequence** |
| --- | --- | --- |
| Cfx P1 | pLMF03 | TTTCATTGTTCCATAAATCAGC |
| Cfx P2 | pLMF03 | TACACCTGTTTTGCATTCTTTT |
| CmpR P1 | RA CH-2 | GGCAAAGACTGAAACAACAAGTCCCT  GTTTCAGAAATAGTGGATTGAG |
| CmpR P2 | RA CH-2 | GCTTCTAGCTCTTCTGCAAGTTCAGGG  GCTCCGAATTTCGGAACCTTC |
| recA up P1 | RA CH-1 | CGATTTGGTAGAGCCTCTTTCAC |
| recA up P2 | RA CH-1 | CTCAATCCACTATTTCTGAAACAGGGA  CTTGTTGTTTCAGTCTTTGCC |
| recA down P1 | RA CH-1 | GAAGGTTCCGAAATTCGGAGCCCCTG  AACTTGCAGAAGAGCTAGAAGC |
| recA down P2 | RA CH-1 | TCGGCATCCATACACTCTATCAC |
| B739_0173 promoter P1 | RA CH-1 | TTTGTTCTTTTAAGTAGTATATCTCCC |
| B739_0173 promoter P1 | RA CH-1 | AGCTGAATAAATTCTTCGCAAAAG |
| B739_0173 coding region P1 | RA CH-1 | GATTAACGGAGTACCTGCATCTAC |
| B739_0173 coding region P2 | RA CH-1 | GACCCATAAGATACAGAAGCGTTG |
